# Cystic hygroma in a female suckling pig: a case report

**DOI:** 10.1186/s40813-022-00285-8

**Published:** 2022-09-30

**Authors:** R. G. Holleboom, D. Bombosch, M. M. H. Wispels, G. Giglia, T. J. Tobias

**Affiliations:** 1 Department of Population Health Sciences, Faculty of Veterinary Medicine, Utrecht University, Yalelaan 7, 3584 CL Utrecht, The Netherlands; 2 Department of Biomolecular Health Sciences, Veterinary Pathology Diagnostic Centre, Faculty of Veterinary Medicine, Utrecht University, Yalelaan 1, 3584 CL Utrecht, The Netherlands

**Keywords:** Lymphatic system, Lymphangioma, Neoplasia, Swine, Neonatal pig, Congenital malformation

## Abstract

**Background:**

Cystic hygromas (lymphangiomas) are rarely reported in various animal species, humans included. A hygroma is a benign congenital malformation of the lymphatic drainage system, presenting itself as a mass consisting of multiple cysts of various sizes with a watery content.

**Case presentation:**

This report describes clinical, ultrasonographic, and post-mortem findings of a cystic hygroma in a suckling pig. The mass was characterized by a few thin-walled cysts, containing clear yellow serous fluid. Histologically, the central cavity was lined by a single layer of squamous cells, supported by a thick fibrous stroma. On immunohistochemistry, scattered lining cells were weakly positive for Factor-VIII, suggesting their possible endothelial origin.

**Conclusions:**

This case report contributes to raising awareness on this condition in pigs allowing early identification in life so that appropriate care can be provided. The case report attributes to science on hygromas in general, as better understanding of pathologic features, the aetiology and appropriate treatment are needed.

## Background

A cystic hygroma is a benign congenital malformation of the lymphatic drainage system, presenting itself as a mass consisting of multiple cysts of various sizes with a serous content [1]. Although a rare condition, cystic hygroma is the most common type of lymphangiomas and has been reported sporadically in various animal species, including horses [2, 3], cattle [4], dogs [5, 6], and recently in pigs as well [7]. Most reports on cystic hygromas are of human cases, in which cystic hygromas are commonly found during prenatal ultrasound investigations or in young infants [8]. In humans, cystic hygromas are mostly reported to occur in the neck, the axillary, and the clavicle region [1, 8], but they can occur anywhere as evidenced by cases described in the abdominal cavity [2, 6].

Although the exact embryonic origin of cystic hygroma is not clarified, it is hypothesized that they result from developmental defects or a cystic malformation of dilated lymphatic channels [1]. In humans, both familial cases and associations with other congenital disorders are reported. Therefore, an underlying genetic cause is suggested by some authors [reviewed by 7]. Treatment is defined case by case depending on the extent, the site of the mass, and the expected complications. In humans, the treatment can comprise of surgical excision, drainage, sclerosant agents as well as conservative management [8, 9]. However, systematic reviews on treatment in humans are lacking. In pigs, little knowledge is present for this condition, and although for commercial pigs, treatment seems incompatible with safe food production standards, treatment may be warranted for pet pigs. In addition, knowledge on the possible genetic basis at the origin of this condition could be beneficial for a safe breeding selection. Pigs and human share many biological similarities, and for this reason development of porcine models of human disease have been suggested. Description of shared spontaneous conditions are needed to study the similarity and differences in their aetiology. In addition, potential porcine models could provide options for comparison of diagnostics and treatment methods in a standardized way. The aim of this case report is to raise awareness in the field of pig health care about the occurrence of cystic hygromas in pigs to result in early recognition and proper diagnosis.

## Clinical presentation

**Fig. 1:**
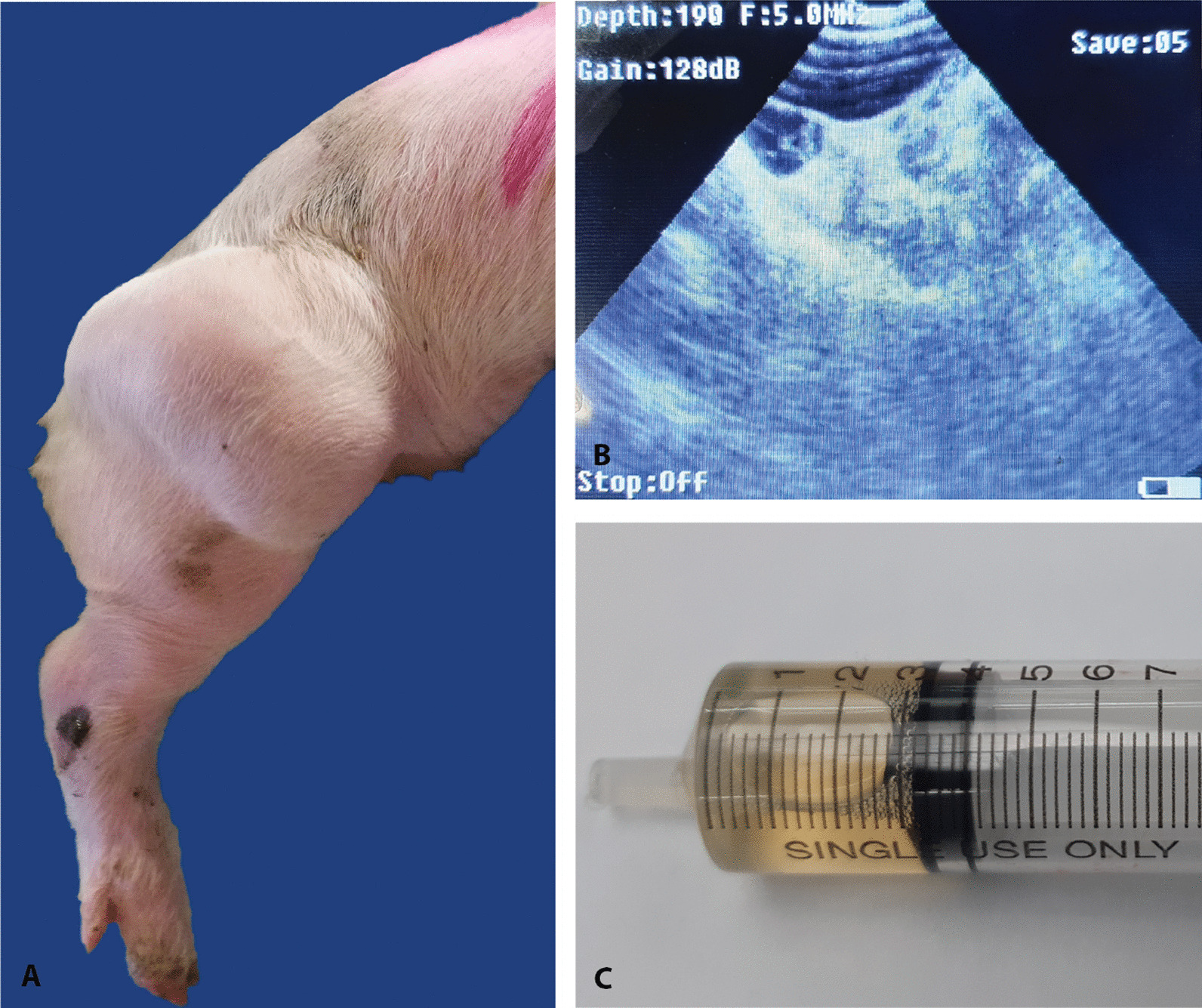
**A** Clinical presentation of the cystic hygroma case, a 10 × 7 cm mass proximal to the right femur. **B** Ultrasonographic imaging of cystic hygroma showing a multi-cystic structure with septations. **C** Aspirated clear and serous fluid from the mass

In October 2021, a 3.5-week-old, farm-bred, female crossbred piglet (Landrace × Yorkshire × Terra (sow) × Large White (boar)) weighing approximately 4 kg was presented with a mass cranial to the right femur (Fig. 1A). The piglet was an offspring to a first parity sow which farrowed 16 life born and 2 mummies 116 days after insemination and was housed in a conventional farrowing crate. It was the only affected pig on the farm and there is no farm history of previous cases. The pig appeared generally healthy and its size and weight were similar to the littermates. The pig’s respiration rate, heart rate and body temperature were all within reference values. No locomotive abnormalities were seen. The mass was first noticed at an age of 2.5 weeks, and according to the animal caretaker it increased in size over time. At the time of clinical examination, the mass was approximately 10 × 7 cm in diameter, soft, painless, with a clear irregular surface and clear demarcation from the surrounding unaffected tissue, the overlying skin was intact. The clinical differential diagnosis included abscessation, hernia, post-traumatic hematoma, and neoplasms.

Explorative ultrasonographic examination, using an ultrasound device with a frequency of 5.0 MHz and a sector probe, revealed a fluid-filled multi-cystic structure with thin internal septations (Fig. 1B). The piglet was euthanized at 3.5 weeks of age and submitted for post-mortem examination after taking into consideration that the condition was incompatible with rearing as a finisher pig and that the pig would likely be condemned at slaughter as suspected of abscessation.

## Pathology and immunohistochemical examination

The day after euthanasia, complete post-mortem examination was performed on the piglet. At the external examination, no abnormalities were present other than the mass proximal to the right femur. Clear, serous fluid was obtained aspirating the content of the mass (Fig. 1C). On cut section at necropsy, the mass appeared expanding into the subcutis of the caudal lateral abdomen and right cranial hip joint region (Fig. 1A, 2A). It appeared as a fluid filled cavity with thin and fragile central septations surrounded by a white and variably thickened firm capsule (0.5–1 mm). No visible communication to other anatomical structures was observed.

**Fig. 2:**
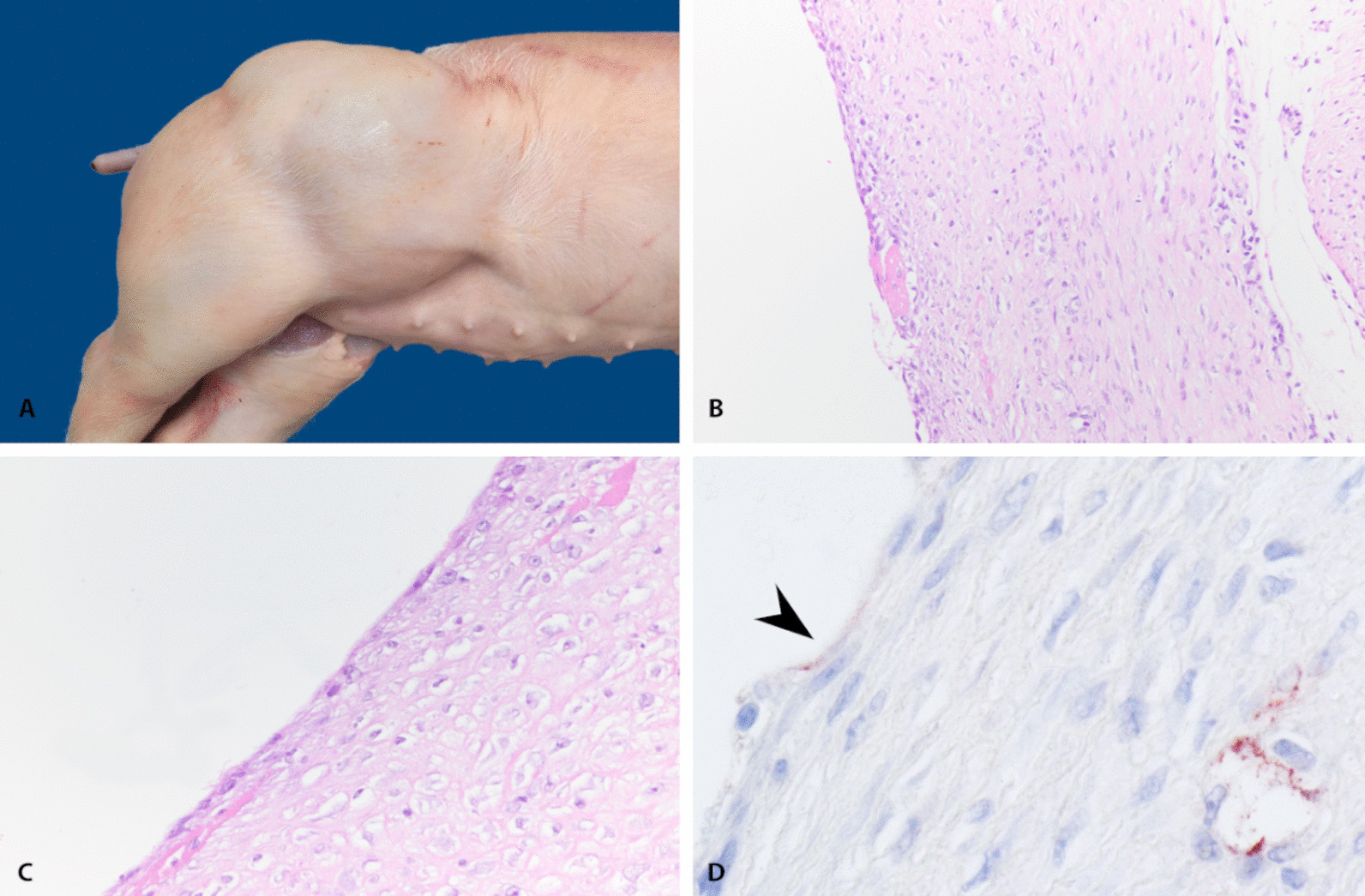
**A**–**D** Macroscopic, histologic and immunohistochemical investigation of the cystic hygroma; **A** a large mass is seen in the right cranial hip joint region. **B** Wall of the cystic mass composed of a peripheral fibrous capsule lined by a simple squamous epithelium (× 20). **C** Closer view on the squamous cells lining in single layer the central cavity (× 40); **D** immunohistochemical positivity for Factor-VIII is seen as week cytoplasmic granular staining in multifocal spindle lining cells (arrowhead) lining the central cavity of the mass and the vessels immersed in the wall (× 40)

Histologically, a well-circumscribed and encapsulated cystic lesion was observed. At the periphery, a thick layer of fibroblasts immersed in abundant eosinophilic material (collagen) was seen (Fig. 2B) few aggregates of mononuclear infiltrating cells represented by a low number of lymphocytes and plasma cells. Lining the central cavity, was a simple layer of squamous cells (endothelium), supported by a thick fibrous stroma. Cells were spindle, up to 20 × 1 µm, with a scant amount of basophilic cytoplasm, poorly defined margins, and a single, oval, and centrally located nucleus with finely coarse chromatin and inapparent nucleoli without features of malignancy (Fig. 2C). Multifocally, Loss of the endothelium was associated with the presence of amorphous to fibrillar and finely beaded, eosinophilic material (fibrin). Based on the macroscopic and histologic findings, a morphological diagnosis of a cystic hygroma was suggested. To confirm the histological type of lining cells, anti-Factor VIII immunohistochemistry was performed. Cells showed a scattered and weak cytoplasmic positivity (Fig. 2D), confirming their possible endothelial origin.

## Discussion

The authors present the findings of clinical and post-mortem examination of a case of cystic hygroma (lymphangioma) in a young female piglet. The diagnosis was made on the typical clinical presentation and the macroscopic and microscopic findings. This case greatly resembles the cases mentioned in the only previous study in pigs, by Letko et al. [7], both in clinical appearance and findings on post-mortem examination.

Regarding the pathogenesis of this lesion, based on the age of the animals affected in our and in the previously published studies (piglets) [7], on the histologic type and the scattered Factor-VII positivity of squamous epithelium lining the cystic structure (endothelium) and the lack of features of malignancy in the proliferating cells (e.g., anisokaryosis and anisocytosis), this lesion seems to very likely be the result of a lymphatic developmental defects (cystic malformation) or a severe dilation of lymphatic channels [1].

Further research into this topic could be valuable for a better understanding of cystic hygromas and lymphangiomas in general, not only in swine but also in other species, including humans. More understanding with regard to the underlying pathological processes and likely causes could provide a basis for developing advanced treatment and prevention options in such cases in any of the species.

Although this case study provides an interesting insight into a rare and under-documented condition, there are limitations. As the rarity and value of the case was not yet appreciated at that time quality and extent of ultrasonographic examinations were limited. For future cases, use of ultrasound or MRI is recommended to visualize the extent and potential communication with other structures of fluid filled cavities in vivo in more detail. In addition, although the fluid content of the mass was not further investigated in this case, cytology or microscopic examination of sediment in addition to protein analyses of the content can provide information about the nature of the content, exudate or transudate, to further narrow down the differential diagnosis. Based on the morphologic and immunohistochemical features, a reactive hygroma developing in the subcutaneous groin region could still not be fully excluded and should be still considered in the list of differential diagnoses. In particular, in the absence of fluid examination additionally supporting the lymphatic origin, a differential might be a trauma-induced damage to the subcutis with necrosis and panniculitis and fluid accumulation forming a reactive fluid-filled cavity (hygroma) surrounded by thick fibrous tissue and sprouting of new blood vessels [10]. In prenatal human cases of lymphangioma, chromosomal alterations of cells derived after aspiration have been investigated to detect associations with other genetic syndromes [11] of which specific ones are predominantly found in either male or females. Therefore defining potential associations with the gender of this case in pigs can be important, but based on Letko et al. [7] and this report no such associations for cystic hygroma in pigs can yet be made.

Euthanizing the piglet gave valuable insight into the structure and makeup of the lesion, however, it limited our ability to assess changes in the lesion over time and during growth and development of the piglet. Spontaneous resolution of cystic hygromas [9] as well as complications such as infection of and bleeding in the cysts have been described in humans [8]. According to the authors, prognosis of the extensive swelling in the pig was deemed poor and conditions in commercial husbandry were favourable for complications, based on which it was decided to euthanise the pig.

As hygromas may occur in body cavities without any external signs, and as infected hygromas can be difficult to differentiate from pure abscesses [1], awareness of this condition in piglets by veterinarians might help setting correct diagnosis, appropriate choice of care dependent on the husbandry situation or timely euthanasia as well as determination of the morbidity and aetiologic factors of cystic hygromas.

## Data Availability

Not applicable.
